# 3-Nitro­benzene-1,2-diamine

**DOI:** 10.1107/S1600536811016825

**Published:** 2011-05-07

**Authors:** Richard Betz, Thomas Gerber

**Affiliations:** a Nelson Mandela Metropolitan University, Summerstrand Campus, Department of Chemistry, University Way, Summerstrand, PO Box 77000, Port Elizabeth 6031, South Africa

## Abstract

The mol­ecule of the title compound, C_6_H_7_N_3_O_2_, a derivative of *o*-phenyl­enediamine, nearly shows non-crystallographic *C*_s_ symmetry. C—C—C angles span the range 116.25 (11)–122.35 (11)°. In the crystal, inter­molecular N—H⋯O and N—H⋯N hydrogen bonds connect mol­ecules into undulating sheets perpendicular to the crystallographic *a* axis. A weak intra­molecular N—H⋯O hydrogen bond is also observed. No π-stacking is observed in the crystal structure.

## Related literature

For the crystal structure of 1,2-diamino­benzene, see: Stalhandske (1981 ▶); Czapik & Gdaniec (2010 ▶). For graph-set analysis of hydrogen bonds, see: Etter *et al.* (1990 ▶); Bernstein *et al.* (1995 ▶). For the use of chelate ligands in coordination chemistry, see: Gade (1998 ▶). For the crystal structures of coordination compounds with rhenium in different oxidation states applying (mixed) oxygen-, nitro­gen- and/or sulfur-containing ligands, see: Chiozzone *et al.* (1999 ▶); Videira *et al.* (2009 ▶); Edwards *et al.* (1998 ▶); Marti *et al.* (2005 ▶); Babich *et al.* (2001 ▶).
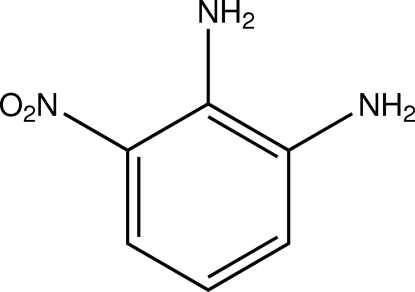


## Experimental

### 

#### Crystal data

C_6_H_7_N_3_O_2_
*M* *_r_* = 153.15
Monoclinic, 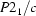

*a* = 13.2854 (5) Å
*b* = 3.7504 (1) Å
*c* = 16.3309 (6) Å
β = 126.208 (2)°
*V* = 656.55 (4) Å^3^
*Z* = 4
Mo *K*α radiation
μ = 0.12 mm^−1^
*T* = 200 K
0.55 × 0.24 × 0.11 mm

#### Data collection

Bruker APEXII CCD diffractometer
6477 measured reflections
1605 independent reflections
1262 reflections with *I* > 2σ(*I*)
*R*_int_ = 0.043

#### Refinement

*R*[*F* ^2^ > 2σ(*F* ^2^)] = 0.040
*wR*(*F* ^2^) = 0.115
*S* = 1.05
1605 reflections
113 parameters
6 restraints
H atoms treated by a mixture of independent and constrained refinement
Δρ_max_ = 0.30 e Å^−3^
Δρ_min_ = −0.17 e Å^−3^

### 

Data collection: *APEX2* (Bruker, 2010 ▶); cell refinement: *SAINT* (Bruker, 2010 ▶); data reduction: *SAINT*; program(s) used to solve structure: *SHELXS97* (Sheldrick, 2008 ▶); program(s) used to refine structure: *SHELXL97* (Sheldrick, 2008 ▶); molecular graphics: *ORTEP-3* (Farrugia, 1997 ▶) and *Mercury* (Macrae *et al.*, 2008 ▶); software used to prepare material for publication: *SHELXL97* and *PLATON* (Spek, 2009 ▶).

## Supplementary Material

Crystal structure: contains datablocks I, global. DOI: 10.1107/S1600536811016825/sj5135sup1.cif

Structure factors: contains datablocks I. DOI: 10.1107/S1600536811016825/sj5135Isup2.hkl

Supplementary material file. DOI: 10.1107/S1600536811016825/sj5135Isup3.cml

Additional supplementary materials:  crystallographic information; 3D view; checkCIF report

## Figures and Tables

**Table 1:** Hydrogen-bond geometry (Å, °)

*D*—H⋯*A*	*D*—H	H⋯*A*	*D*⋯*A*	*D*—H⋯*A*
N1—H711⋯O1^i^	0.89 (1)	2.41 (2)	3.1257 (14)	138 (2)
N2—H721⋯N1^ii^	0.88 (1)	2.26 (1)	3.0800 (16)	156 (2)
N2—H722⋯O1	0.88 (1)	1.98 (1)	2.6084 (14)	127 (1)
N2—H722⋯O1^iii^	0.88 (1)	2.55 (2)	3.1416 (16)	126 (1)

Symmetry codes: (i) 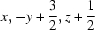; (ii) 
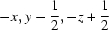
; (iii) 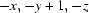.

**Fig. 1.**
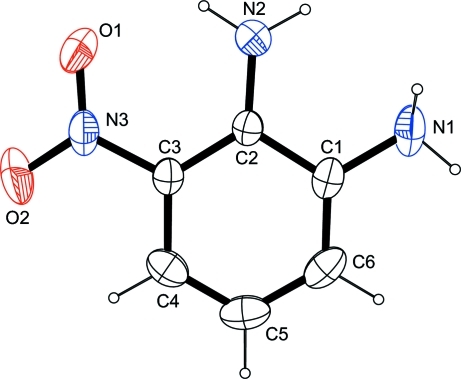
The molecular structure of the title compound, with atom labels and anisotropic displacement ellipsoids (drawn at 50% probability level).

**Fig. 2.**
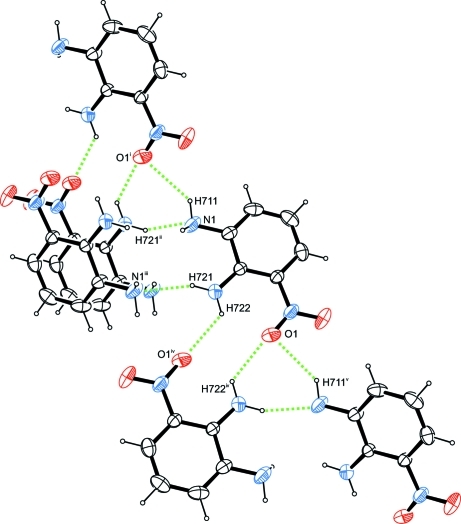
Intermolecular contacts, viewed approximately along [010]. Symmetry operators: ix, -y + 3/2, z + 1/2; ii -x, y + 1/2, -z + 1/2; iii -x, y - 1/2, -z + 1/2; iv -x, -y + 1, -z; vx, -y + 3/2, z - 1/2.

**Fig. 3.**
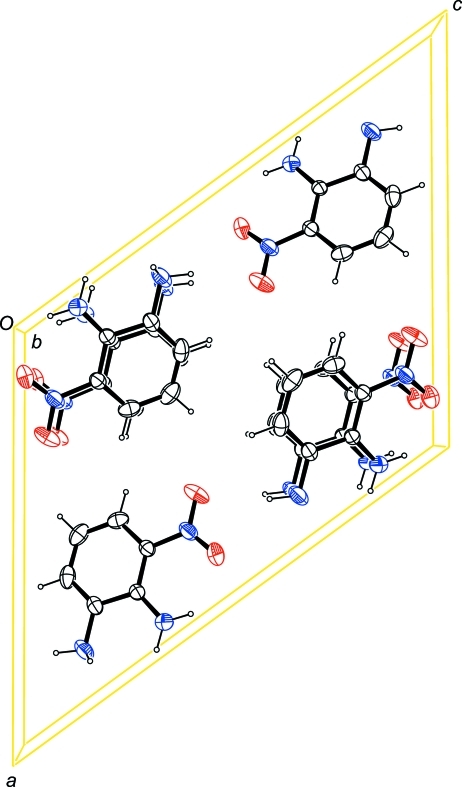
Molecular packing of the title compound, viewed along [0 1 0] (anisotropic displacement ellipsoids drawn at 50% probability level).

## supplementary crystallographic information

### Comment

Chelate ligands have found widespread use in coordination chemistry due to the enhanced thermodynamic stability of resultant coordination compounds in relation to coordination compounds exclusively applying comparable monodentate ligands (Gade 1998). Combining different sets of donor atoms in one chelate ligand molecule, a probe for testing and accomodating metal centers of different Lewis acidities is at hand. For the crystal structures of coordination compounds with rhenium in different oxidation states applying (mixed) oxygen-, nitrogen- and/or sulfur-containing ligands, see: Chiozzone *et al.* (1999); Videira *et al.* (2009); Edwards *et al.* (1998); Marti *et al.* (2005); Babich *et al.* (2001).  The title compound, which offers two amino and one nitro group in close proximity to each other, seemed particularily interesting in this aspect. To enable comparative studies with the crystal structures of envisioned coordination compounds, the structure of the free ligand was determined. The crystal structure of 1,2-diaminobenzene is apparent in the literature (Stalhandske 1981, Czapik & Gdaniec 2010).

Intracyclic angles cover a range of 116–122 ° with the smallest angle present on the C-atom in between the C-atoms bearing the nitro as well as an amino group. The nitro group is nearly completely in plane with the aromatic system. The least-squares planes defined by their respective atoms intersect at an angle of only 3.93 (18) ° (Fig. 1).

Except for one of the H-atoms of the amino group in *meta*-position to the nitro group, all of the hydrogen atoms of the amino groups participate in hydrogen bonds in the crystal structure. While one of the O-atoms of the nitro group acts as twofold acceptor, the second one does not take part in this type of intermolecular contacts. In terms of graph-set analysis, (Etter *et al.* 1990, Bernstein *et al.* 1995), the descriptor for the hydrogen bonding system on the unitary level is *C*^1^_1_(5)*C*^1^_1_(7)*R*^2^_2_(12). In total, the molecules are connected to waved sheets perpendicular to the crystallographic *a*-axis. π-stacking is not observed in the crystal structure of the title compound (Fig. 2).

The molecular packing is shown in Figure 3.

### Experimental

The compound was obtained commercially (Aldrich). Crystals suitable for the X-ray diffraction study were obtained upon recrystallization from ethanol.

### Refinement

Carbon-bound H-atoms were placed in calculated positions (C—H 0.95 Å) and were included in the refinement in the riding model approximation, with *U*(H) set to 1.2*U*_eq_(C). The H-atoms of the amine groups were located on a difference Fourier map, and their N—H distances as well as their H–N–H angles were refined using *DFIX* instructions with one common free variable, with their *U*(H) set to 1.5*U*_eq_(N).

### Crystal data

**Table:**

C_6_H_7_N_3_O_2_	*F*(000) = 320
*M**_r_* = 153.15	*D*_x_ = 1.549 Mg m^−^^3^
Monoclinic, *P*2_1_/*c*	Mo *K*α radiation, λ = 0.71073 Å
Hall symbol: -P 2ybc	Cell parameters from 3115 reflections
*a* = 13.2854 (5) Å	θ = 2.5–28.2°
*b* = 3.7504 (1) Å	µ = 0.12 mm^−^^1^
*c* = 16.3309 (6) Å	*T* = 200 K
β = 126.208 (2)°	Rod, red
*V* = 656.55 (4) Å^3^	0.55 × 0.24 × 0.11 mm
*Z* = 4	

### Data collection

**Table:**

Bruker APEXII CCD diffractometer	1262 reflections with *I* > 2σ(*I*)
Radiation source: fine-focus sealed tube	*R*_int_ = 0.043
graphite	θ_max_ = 28.3°, θ_min_ = 3.1°
φ and ω scans	*h* = −17→17
6477 measured reflections	*k* = −4→4
1605 independent reflections	*l* = −21→21

### Refinement

**Table:**

Refinement on *F*^2^	Primary atom site location: structure-invariant direct methods
Least-squares matrix: full	Secondary atom site location: difference Fourier map
*R*[*F*^2^ > 2σ(*F*^2^)] = 0.040	Hydrogen site location: inferred from neighbouring sites
*wR*(*F*^2^) = 0.115	H atoms treated by a mixture of independent and constrained refinement
*S* = 1.05	*w* = 1/[σ^2^(*F*_o_^2^) + (0.055*P*)^2^ + 0.1822*P*] where *P* = (*F*_o_^2^ + 2*F*_c_^2^)/3
1605 reflections	(Δ/σ)_max_ < 0.001
113 parameters	Δρ_max_ = 0.30 e Å^−^^3^
6 restraints	Δρ_min_ = −0.17 e Å^−^^3^

### Fractional atomic coordinates and isotropic or equivalent isotropic displacement parameters (Å^2^)

**Table:**

	*x*	*y*	*z*	*U*_iso_*/*U*_eq_	
O1	0.13915 (10)	0.7321 (4)	0.03335 (7)	0.0524 (3)	
O2	0.30745 (12)	1.0010 (4)	0.07783 (10)	0.0687 (4)	
N1	0.13706 (12)	0.8731 (4)	0.34249 (9)	0.0419 (3)	
H711	0.1778 (16)	0.888 (5)	0.4092 (10)	0.063*	
H712	0.1066 (16)	0.649 (4)	0.3250 (14)	0.063*	
N2	0.07013 (10)	0.7189 (3)	0.15331 (8)	0.0346 (3)	
H721	0.0209 (14)	0.662 (5)	0.1708 (12)	0.052*	
H722	0.0458 (15)	0.663 (5)	0.0919 (10)	0.052*	
N3	0.23536 (11)	0.9000 (3)	0.09715 (8)	0.0382 (3)	
C1	0.22068 (11)	0.9559 (3)	0.31843 (9)	0.0289 (3)	
C2	0.18152 (10)	0.8773 (3)	0.21809 (8)	0.0241 (3)	
C3	0.26459 (10)	0.9743 (3)	0.19499 (8)	0.0268 (3)	
C4	0.37907 (11)	1.1429 (3)	0.26574 (11)	0.0349 (3)	
H4	0.4329	1.2060	0.2477	0.042*	
C5	0.41227 (12)	1.2152 (4)	0.36057 (10)	0.0397 (3)	
H5	0.4895	1.3289	0.4091	0.048*	
C6	0.33235 (12)	1.1214 (4)	0.38622 (9)	0.0374 (3)	
H6	0.3561	1.1739	0.4524	0.045*	

### Atomic displacement parameters (Å^2^)

**Table:**

	*U*^11^	*U*^22^	*U*^33^	*U*^12^	*U*^13^	*U*^23^
O1	0.0500 (6)	0.0787 (8)	0.0285 (5)	−0.0038 (6)	0.0231 (5)	−0.0108 (5)
O2	0.0701 (8)	0.1063 (11)	0.0630 (7)	−0.0052 (7)	0.0575 (7)	0.0026 (7)
N1	0.0512 (7)	0.0525 (8)	0.0371 (6)	0.0146 (6)	0.0344 (6)	0.0103 (6)
N2	0.0294 (5)	0.0433 (7)	0.0331 (5)	−0.0028 (4)	0.0195 (5)	−0.0030 (5)
N3	0.0430 (6)	0.0493 (7)	0.0343 (6)	0.0089 (5)	0.0294 (5)	0.0054 (5)
C1	0.0353 (6)	0.0286 (6)	0.0263 (5)	0.0121 (5)	0.0201 (5)	0.0066 (5)
C2	0.0261 (5)	0.0237 (5)	0.0244 (5)	0.0061 (4)	0.0160 (4)	0.0031 (4)
C3	0.0299 (6)	0.0276 (6)	0.0264 (5)	0.0052 (4)	0.0185 (5)	0.0035 (4)
C4	0.0300 (6)	0.0283 (6)	0.0472 (7)	0.0024 (5)	0.0233 (6)	0.0037 (5)
C5	0.0305 (6)	0.0294 (7)	0.0404 (7)	0.0011 (5)	0.0106 (5)	−0.0050 (5)
C6	0.0433 (7)	0.0331 (7)	0.0249 (6)	0.0114 (5)	0.0140 (5)	−0.0011 (5)

### Geometric parameters (Å, °)

**Table:**

O1—N3	1.2420 (16)	C1—C6	1.3681 (18)
O2—N3	1.2318 (15)	C1—C2	1.4268 (15)
N1—C1	1.4142 (16)	C2—C3	1.4088 (15)
N1—H711	0.887 (12)	C3—C4	1.4041 (17)
N1—H712	0.903 (12)	C4—C5	1.364 (2)
N2—C2	1.3462 (15)	C4—H4	0.9500
N2—H721	0.880 (12)	C5—C6	1.397 (2)
N2—H722	0.878 (12)	C5—H5	0.9500
N3—C3	1.4313 (15)	C6—H6	0.9500
			
C1—N1—H711	108.5 (12)	C3—C2—C1	116.25 (11)
C1—N1—H712	113.3 (12)	C4—C3—C2	122.35 (11)
H711—N1—H712	106.4 (15)	C4—C3—N3	117.01 (11)
C2—N2—H721	122.3 (11)	C2—C3—N3	120.64 (11)
C2—N2—H722	119.6 (11)	C5—C4—C3	119.38 (12)
H721—N2—H722	118.0 (14)	C5—C4—H4	120.3
O2—N3—O1	120.77 (12)	C3—C4—H4	120.3
O2—N3—C3	119.13 (12)	C4—C5—C6	119.81 (12)
O1—N3—C3	120.09 (10)	C4—C5—H5	120.1
C6—C1—N1	121.95 (11)	C6—C5—H5	120.1
C6—C1—C2	120.56 (11)	C1—C6—C5	121.65 (12)
N1—C1—C2	117.41 (11)	C1—C6—H6	119.2
N2—C2—C3	125.10 (10)	C5—C6—H6	119.2
N2—C2—C1	118.65 (10)		
			
C6—C1—C2—N2	−179.04 (11)	O1—N3—C3—C4	175.60 (12)
N1—C1—C2—N2	−2.26 (17)	O2—N3—C3—C2	177.07 (12)
C6—C1—C2—C3	0.96 (17)	O1—N3—C3—C2	−3.77 (19)
N1—C1—C2—C3	177.74 (10)	C2—C3—C4—C5	0.29 (19)
N2—C2—C3—C4	179.27 (11)	N3—C3—C4—C5	−179.07 (11)
C1—C2—C3—C4	−0.73 (17)	C3—C4—C5—C6	−0.04 (19)
N2—C2—C3—N3	−1.39 (19)	N1—C1—C6—C5	−177.40 (12)
C1—C2—C3—N3	178.60 (10)	C2—C1—C6—C5	−0.78 (19)
O2—N3—C3—C4	−3.56 (19)	C4—C5—C6—C1	0.3 (2)

### Hydrogen-bond geometry (Å, °)

**Table:**

*D*—H···*A*	*D*—H	H···*A*	*D*···*A*	*D*—H···*A*
N1—H711···O1^i^	0.89 (1)	2.41 (2)	3.1257 (14)	138.(2)
N2—H721···N1^ii^	0.88 (1)	2.26 (1)	3.0800 (16)	156.(2)
N2—H722···O1	0.88 (1)	1.98 (1)	2.6084 (14)	127.(1)
N2—H722···O1^iii^	0.88 (1)	2.55 (2)	3.1416 (16)	126.(1)

Symmetry codes: (i) *x*, −*y*+3/2, *z*+1/2; (ii) −*x*, *y*−1/2, −*z*+1/2; (iii) −*x*, −*y*+1, −*z*.
